# Public health impacts of secondary particulate formation from aromatic hydrocarbons in gasoline

**DOI:** 10.1186/1476-069X-12-19

**Published:** 2013-02-20

**Authors:** Katherine von Stackelberg, Jonathan Buonocore, Prakash V Bhave, Joel A Schwartz

**Affiliations:** 1 Harvard Center for Risk Analysis, 401 Park Drive, Landmark 404J, Boston, MA 02215, USA; 2 National Exposure Research Laboratory, Office of Research & Development, U.S. Environmental Protection Agency, 109 T.W. Alexander Dr. Research Triangle Park, Durham, NC, 27711, USA

**Keywords:** Aromatic hydrocarbons, Secondary organic aerosol (SOA), Secondary particulate, Social cost, Gasoline

## Abstract

**Background:**

Aromatic hydrocarbons emitted from gasoline-powered vehicles contribute to the formation of secondary organic aerosol (SOA), which increases the atmospheric mass concentration of fine particles (PM_2.5_). Here we estimate the public health burden associated with exposures to the subset of PM_2.5_ that originates from vehicle emissions of aromatics under business as usual conditions.

**Methods:**

The PM_2.5_ contribution from gasoline aromatics is estimated using the Community Multiscale Air Quality (CMAQ) modeling system and the results are compared to ambient measurements from the literature. Marginal PM_2.5_ annualized concentration changes are used to calculate premature mortalities using concentration-response functions, with a value of mortality reduction approach used to monetize the social cost of mortality impacts. Morbidity impacts are qualitatively discussed.

**Results:**

Modeled aromatic SOA concentrations from CMAQ fall short of ambient measurements by approximately a factor of two nationwide, with strong regional differences. After accounting for this model bias, the estimated public health impacts from exposure to PM_2.5_ originating from aromatic hydrocarbons in gasoline lead to a central estimate of approximately 3800 predicted premature mortalities nationwide, with estimates ranging from 1800 to over 4700 depending on the specific concentration-response function used. These impacts are associated with total social costs of $28.2B, and range from $13.6B to $34.9B in 2006$.

**Conclusions:**

These preliminary quantitative estimates indicate particulates from vehicular emissions of aromatic hydrocarbons demonstrate a nontrivial public health burden. The results provide a baseline from which to evaluate potential public health impacts of changes in gasoline composition.

## Background

Field studies suggest 10% - 60% of fine particulate matter (PM_2.5_) is comprised of organic compounds [1-3]. This material may be directly emitted to the atmosphere (primary) or formed from the gas-phase oxidation of hydrocarbon molecules and subsequent absorption into the condensed phase (secondary). The latter portion, referred to as secondary organic aerosol (SOA), is a major contributor to the PM_2.5_ burden in both urban and rural atmospheres [4-8], which contributes to a range of adverse health effects [9-12], visibility reduction [13,14], and global climate change [15-17].

In the atmosphere, SOA can originate from both anthropogenic (e.g., solvent use, mobile sources) and biogenic (e.g., forests) sources. Of the anthropogenic precursors, conventional wisdom is that aromatic hydrocarbons are among the most efficient at forming SOA [18,19]. Table 1 lists several empirical studies that estimated the contribution of SOA precursors to observed PM_2.5_ concentrations. These studies show that aromatics typically contribute between 0.1 to 0.45 μg/m^3^ to observed PM_2.5_ concentrations [20-23].

**Table 1:** Studies evaluating the contribution of aromatic hydrocarbons to SOA

**Reference**	**Description**	**Source apportionment**	**Concentrations (μg/m**^**3**^**)**
[20]	Contribution of primary and secondary sources of OC to PM_2.5 _in a small subset of Southeastern Aerosol Research and Characterization (SEARCH) network samples	(2,3-hydroxy-4-oxopentanoic acid used as a chemical tracer for aromatic SOA	0.10 to 0.45 across 4 sampling locations

[21]	Contribution of primary and secondary sources of OC to PM_2.5 _in five midwestern United States cities year–round: East St. Louis, IL Detroit, MI Cincinnati, OH Bondville, IL and Northbrook, IL	2,3-hydroxy-4-oxopentanoic acid used as a chemical tracer for aromatic SOA	Bondville: 0.09 - 0.25; Northbrook: 0.06 - 0.21; Cincinnati: 0.02 - 0.29; Detroit: 0.07 - 0.33; East St. Louis: 0.06 - 0.26

[22]	Contribution of primary and secondary sources of OC to PM_2.5 _in 2006 in Research Triangle Park, NC over the course of a year	2,3-hydroxy-4-oxopentanoic acid used as a chemical tracer for aromatic SOA	average = 0.1, stdev = 0.09, min = 0.02, max = 0.36, n = 33

[33]	Positive matrix factorization of organic marker measurements to estimate primary and secondary components of organic aerosol	SOA from motor vehicles contribute 11% of total organic aerosols	method is not quantitative
[23]	Contribution of primary and secondary sources of OC to PM_2.5 _in July-August 2007 in Cleveland, OH, Detroit, MI and LA, CA	2,3-hydroxy-4-oxopentanoic acid used as a chemical marker for aromatic SOA, using a different analytical method than [20-22]	0.05 - 1.1 in the midwest; 0.95 - 1.61 in CA

A series of sunlight-irradiated, smog-chamber experiments conducted in the 1980's suggested that the PM_2.5_ formation potential of whole gasoline vapor can be accounted for solely in terms of the aromatic fraction of the fuel [18]. More recent chamber studies show that SOA yields measured under NOx-limited conditions greatly exceed formation under NOx-rich conditions, and that SOA yields under NOx-rich conditions are even greater than were observed previously [24]. Evidence is growing that aromatics in gasoline exhaust are among the most efficient secondary organic matter precursors [19,25]. While the relative abundance of primary and secondary organic matter is the subject of ongoing debate [26], air quality models are continually updated to keep up with the latest scientific knowledge [27,28].

In the United States, gasoline-powered vehicles are the largest source of aromatic hydrocarbons to the atmosphere [29]. Most gasoline formulations consist of approximately 20% aromatic hydrocarbons [30], which are used in place of lead to boost octane. Therefore, it has been suggested that removal of aromatics could reduce SOA concentrations and yield a substantial public health benefit [31]. The issue is complicated by the fact that any change to fuel composition will affect vehicular emissions of various pollutants (e.g., hydrocarbons, carbon monoxide, oxides of nitrogen [NOx], primary PM_2.5_) which, in turn, will react in the atmosphere to produce a different mix of pollutants that may have adverse effects (e.g., [32]). However, a number of studies have noted that gas-phase vehicle emissions lead to a substantial fraction of observed SOA [33]. For example, a source apportionment study of SOA formation during a severe photochemical smog event in Los Angeles found that gasoline engines represented the single-largest anthropogenic source of SOA [34].

The purpose of this study is to estimate the public health impacts and social costs associated with exposure to SOA from vehicular emissions of aromatic hydrocarbons. This analysis provides a baseline case to explore the magnitude of the issue and against which to evaluate the cost and impacts of potential substitutes for aromatics. The next section describes the methods for the analysis, followed by results and a concluding discussion.

## Methods

Predicted secondary PM_2.5_ concentrations attributable to single-ringed aromatic hydrocarbons are estimated for a baseline year (2006) using the Community Multiscale Air Quality model version 5.0 (CMAQv5.0). Given that air quality models are known to underestimate anthropogenic SOA formation [19,26,35], our results are compared to available data to estimate scaling factors for adjusting the model results. Adjusted PM_2.5_ concentrations are then used in the US EPA Benefits and Mapping Program v4.0 (BenMAP) model to estimate morbidity health and mortality outcomes associated with exposure to these concentrations across the lower 48 states [36].

### Exposure concentrations

The CMAQ model is among the most widely used air quality models, with 3000+ registered users in 100 different countries (http://www.cmaq-model.org). Federal and State regulatory agencies use CMAQ for policy analyses and for routine air quality forecasting [37]. The model provides a means for quantitatively evaluating the impact of air quality management policies prior to implementation. This analysis relied on CMAQv5.0 with the Carbon Bond 2005 (CB05) chemical mechanism, which includes a fairly comprehensive list of precursors that lead to SOA formation via both gas- and aqueous-phase oxidation processes, as well as particle-phase reactions [27].

Air quality model simulations based on CMAQv5.0 are used to estimate the total concentration of SOA from all single-ring aromatic compounds (e.g., benzene, toluene, xylenes) in uniform 12km grid cells across the lower 48 states for a baseline year (2006).

### Potential underestimates in predictions of SOA formation

Although CMAQv5.0 contains updated algorithms and processes for predicting SOA formation, evidence suggests that the model may still underestimate secondary PM_2.5_ concentrations [27,28,38], particularly during the summer [37]. Experiments conducted at Carnegie Mellon University to study SOA formation from the photooxidation of toluene suggest significantly larger SOA production than parameterizations employed in current air-quality models [39].

Using an organic tracer-based source apportionment approach, independently conducted research over the last five years provides increasing evidence that aromatic hydrocarbons in gasoline contribute, depending on the specific region, approximately 0.1 to 0.45 μg/m^3^ of PM [20-23].

Given our objective to estimate the public health impact of aromatic SOA, CMAQv5.0 model results must be adjusted to reflect any biases specific to this PM_2.5_ component. Monthly-averaged model results are compared against empirical estimates of aromatic SOA concentrations derived from ambient measurements of 2,3-dihydroxy-4-oxopentanoic acid collected at twelve locations across the U.S. ([20-23,40]. We develop region-specific regression relationships between modeled CMAQ values and measured concentrations in μg of carbon per m^3^ and use these to adjust the model results prior to estimating health effects. Regions are based on standard US census designations consistent with the census designations in subsequent linked models (http://www.eia.gov/emeu/mecs/mecs2002/census.html). We develop a mixed model with a random slope for each region, as there is some indication that slopes should vary by region. For example, Hildebrandt *et al.*[39] report elevated SOA yields from toluene under high UV intensity, low-NO_x_ conditions, and lower temperatures, relative to the parameters used typically in models. Therefore, the slope might be low in California where there is a lot of NO_x_ and high in the Midwest and East where ambient temperatures remain relatively low. The overall fixed effect and region-specific random effects models are developed using REML in R (http://www.r-project.org/) based on the following equation:

(1) $$\begin{array}{l} \text{Formula}:\;\text{SOA}∼\text{CMAQv}5.0\\ \;+\left(\text{CMAQv}5.0|\text{region}\right) \end{array}$$

### SOA formation from aromatic hydrocarbons in gasoline

SPECIATE, a US EPA database, includes a large repository of volatile organic compound (VOC) speciation profiles of air pollution emission sources [29]. We use these source profiles in conjunction with the National Emissions Inventory (from the year 2005) for VOCs to estimate the nationwide proportion of aromatic VOCs attributable to emissions from gasoline vehicles. We rank order all sources of aromatic VOCs to quantify the contribution to total emissions specifically from gasoline-based sources.

### Health and mortality impacts

The BenMAP model was used to estimate resulting health impacts associated with exposures to the change in PM_2.5_ concentrations attributable to aromatic hydrocarbons from gasoline vehicles modeled by the process described above. The BenMAP model is widely used by regulatory agencies to quantify and monetize potential health impacts associated with changes in air quality, and contains concentration-response functions for various pollutants, including PM_2.5_, census data and population projections, and baseline mortality and morbidity rates for the lower 48 United States. Concentration response functions incorporated in BenMAP are based on published studies incorporating different assumptions regarding potential thresholds and observed slopes between concentrations and responses.

Four studies are included in this analysis [12,41-43]. Two major cohort studies are generally thought to provide estimates regarded as most robust and applicable to the general population, with the Harvard Six Cities Study publications reporting central estimates of an approximate 1.2-1.6% increase in all-cause mortality per μg/m^3^ increase in annual average PM_2.5_[42] and the American Cancer Society studies reporting estimates of approximately 0.4-0.6% [12], with higher estimates when exposure characterization was more spatially refined [41]. Within the expert elicitation study [43] (Industrial Economics, Inc. 2006) the median concentration-response function across experts was approximately 1%, midway between these cohort estimates, with a median 5^th^ percentile of 0.3% and a median 95^th^ percentile of 2.0%. The EPA Science Advisory Board external Advisory Committee on Clean Air Act Compliance Analysis recommended developing a distribution with the Pope and Laden studies at the 25^th^ and 75^th^ percentiles, respectively, leading to a mean of the new distribution close to the mean of the central estimates of both Pope and Laden. This generally will be consistent with the distribution identified in the expert elicitation, as recommended by EPA’s Science Advisory Board [44]. BenMAP applies these functions to the baseline mortality rate and the number of people potentially exposed by census tract. BenMAP provides distributions of premature mortality estimates based on the uncertainty in the concentration-response functions. That is, the 5^th^ and 95^th^ percentiles in the results are based on the distributions for concentration-response functions only.

### Monetized estimates of premature mortality

Monetized estimates of premature mortality are based on regulatory estimates of the value of mortality risk as defined by the U.S. EPA [45]. This estimate is based on research in which people are asked how much they would pay for consumer products (such as water filters) that reduce risk or alternatively, that examine how much more employers have to pay employees (adjusting for age, education, experience, etc.) to compensate for taking an increased risk of accidental death. Hence this estimate is not a price on a life, but a price of risk reduction. For convenience it is converted into what was referred to as a value of a statistical life and is now referred to as the value of mortality risk. The implication is if people are willing to pay $X for a reduction in risk of 1 in 10,000, than reducing risk in enough people to produce, on average, one fewer death would be worth 10,000 X dollars. The U.S. EPA recommends a value of $7.4M in 2006 dollars [45] based on over 30 labor market and contingent valuation studies.

## Results

### CMAQv5.0 modeling results compared to measurements

Additional file 1: Table S1 compiles measurement-based estimates of aromatic SOA collected at twelve locations between 2004 and 2010. Concentrations reach as high as 0.41 μgC/m^3^ during the summer in Cincinnati, with a median value of 0.14 μgC/m^3^ across all 77 samples. In contrast, the CMAQv5.0 model results from the corresponding 12 km grid cells and averaged over the appropriate month in 2006 show a maximum value of 0.13 and a median of 0.052 μgC/m^3^ (see Additional file 1: Table S1). This systematic bias in the model results warrants some adjustment of the CMAQv5.0 output before it is used in the BenMAP calculations. The mixed model obtained by regressing observations against the CMAQv5.0 results are shown in Table 2. The slopes do differ by region, with the highest slopes observed in the East and Midwest. Aggregated up to the national level, unadjusted CMAQ results predict a nationwide average concentration of 0.045 μg/m^3^, which increases to 0.17 following the adjustment, a factor of approximately 3.8.

**Table 2:** **Regression relationships developed by region to adjust CMAQv5.0 results based on data in Additional file****1****: Table S1**

	**Value**	**Standard error**	**T-value**	**P-value**
**Overall estimate and slope**				
Intercept	0.01875	0.16	-0.69	0.49
CMAQv5.0	1.896	2.34	1.99	0.05
**Random effects by region**				
Region	Intercept	CMAQv5.0		
Midwest/East	0	1.12		
South	0	-0.269		
West	0	-0.856		
**Final equations used to adjust original CMAQv5.0 results**				
Midwest/East	SOA = 0.01875 + 3.016*CMAQv5.0			
South	SOA = 0.01875 + 1.627*CMAQv5.0			
West	SOA = 0.01875 + 1.04*CMAQv5.0			

Model based on Equation 1.

Linear mixed model fit by REML.

Formula: SOA ~ CMAQv5.0 + (CMAQv5.0 | region).

### Predicted PM_2.5_ concentrations from aromatic hydrocarbons in gasoline

Source-specific speciation of total VOC in the 2005 National Emissions Inventory reveals that the U.S. emissions of single-ring aromatic hydrocarbons are 3.6 million tons per year, of which 69% are from gasoline-powered vehicles [29] as shown in Table 3. A source-by-source breakdown of all aromatic hydrocarbon emissions is provided in Additional file 2: Table S2. To subtract the contribution of other emission sources (e.g., solvent usage, diesel exhaust) from our calculations, the adjusted aromatic SOA concentrations from CMAQv5.0 are multiplied by 0.69.

**Table 3:** National emissions inventory of single-ring aromatic hydrocarbons

**Source**	**Aromatic VOC (ton/yr)**	**% of total**
**Gasoline**	**2,491,313**	**69%**
Solvent usage	518,334	14%
Diesel	25,436	1%
Other	573,679	16%
Total	3,608,762	100%

Note:

This information was obtained by combining VOC emissions from the 2005 National Emissions Inventory with speciation profiles from the SPECIATE database. See Additional file 2: Table S2.

Spatial patterns of aromatic emissions are similar across sources. After gasoline, the next highest source of aromatics is solvent usage, and Reff *et al.*[46] show that the spatial pattern of solvent usage is similar to gasoline, that is, occurs predominantly in urban areas. In addition, most major refineries are also in close proximity to urban areas.

### Adjusted CMAQv5.0 results

**Figure 1:**
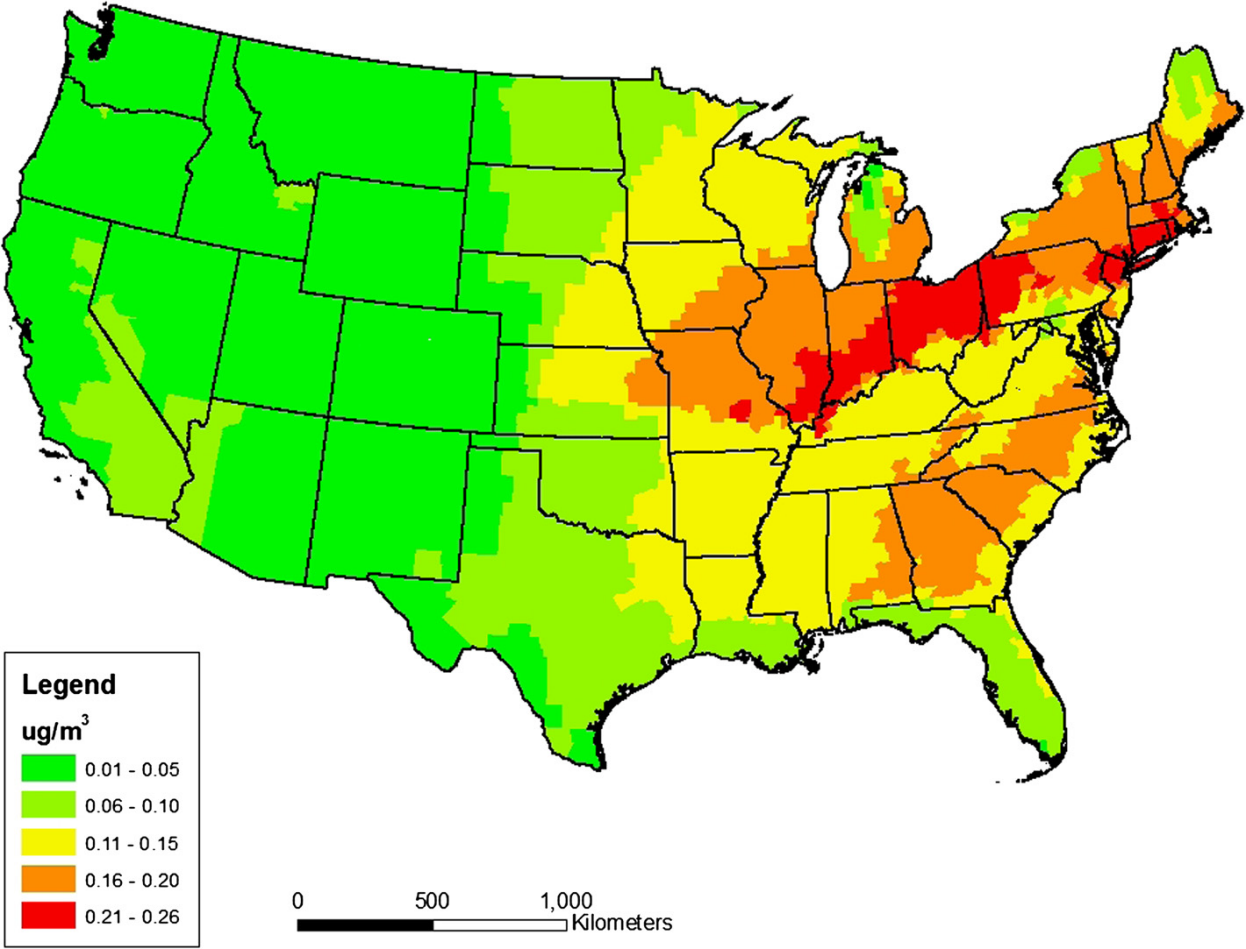
**Annual average PM**_**2.5 **_**concentrations attributed to aromatic emissions from gasoline vehicles, after accounting for region-specific CMAQ model biases and subtracting aromatic contributions from other VOC sources.**

Figure 1 shows the final nationwide distribution of annual average PM_2.5_ concentrations attributable to aromatic hydrocarbons emitted from gasoline vehicles, after applying all the adjustments to the CMAQv5.0 output described above. The nationwide average concentration based on the average predicted value for each state is approximately 0.17 μg/m^3^ (standard deviation = 0.06 μg/m^3^; minimum = 0.03 μg/m^3^, maximum = 0.3 μg/m^3^) and ranges from 0.013 to greater than 0.6 μg/m^3^ at the county level. On a statewide basis, Table 4 shows the rank ordered concentrations by state, with Connecticut, Rhode Island, Ohio, New York, New Jersey and Indiana at or exceeding 0.2 μg/m^3^ statewide.

**Table 4:** **State-wide annual average estimates of PM**_**2.5 **_**attributed to aromatic SOA from gasoline emissions**

**State**	**Predicted PM**_**2.5 **_**concentration (μg/m**^**3**^**)**
CT	0.23
RI	0.23
OH	0.21
NY	0.21
NJ	0.20
IN	0.20
MA	0.19
NH	0.18
IL	0.17
PA	0.17
MO	0.17
MI	0.16
SC	0.16
NC	0.16
GA	0.16
VT	0.15
IA	0.15
WI	0.15
ME	0.14
KY	0.14
DE	0.14
TN	0.14
AL	0.14
WV	0.13
VA	0.13
MS	0.13
KS	0.12
DC	0.12
MD	0.12
AR	0.11
MN	0.11
NE	0.11
OK	0.09
LA	0.09
SD	0.09
TX	0.08
ND	0.08
FL	0.08
NV	0.05
AZ	0.05
CA	0.04
ID	0.04
MT	0.03
UT	0.03
WY	0.03
OR	0.03
WA	0.03
NM	0.03
CO	0.03

### BenMAP modeling results

**Figure 2:**
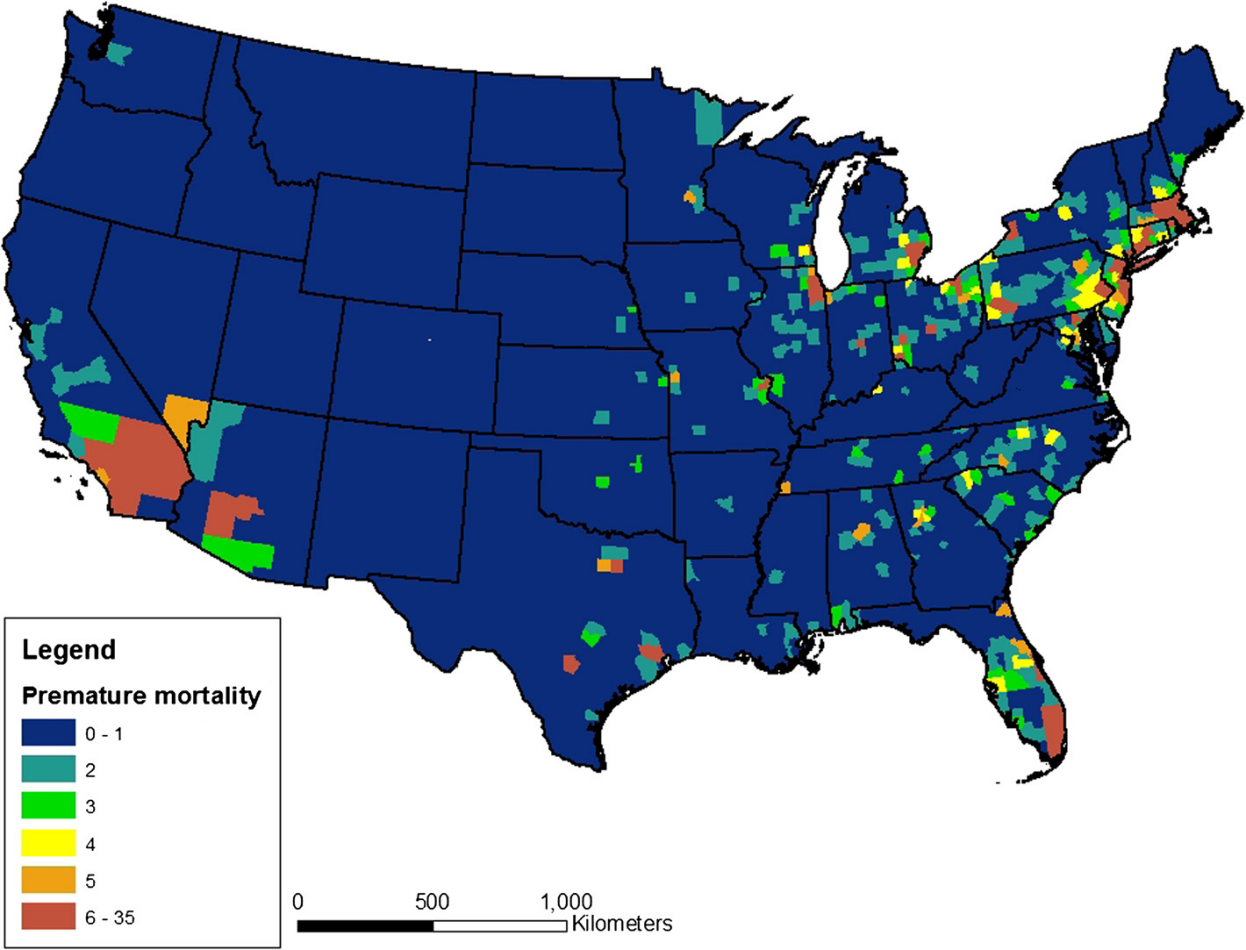
Estimated cases of premature mortality per year in each U.S. county based on the consensus expert elicitation concentration-response function.

**Figure 3:**
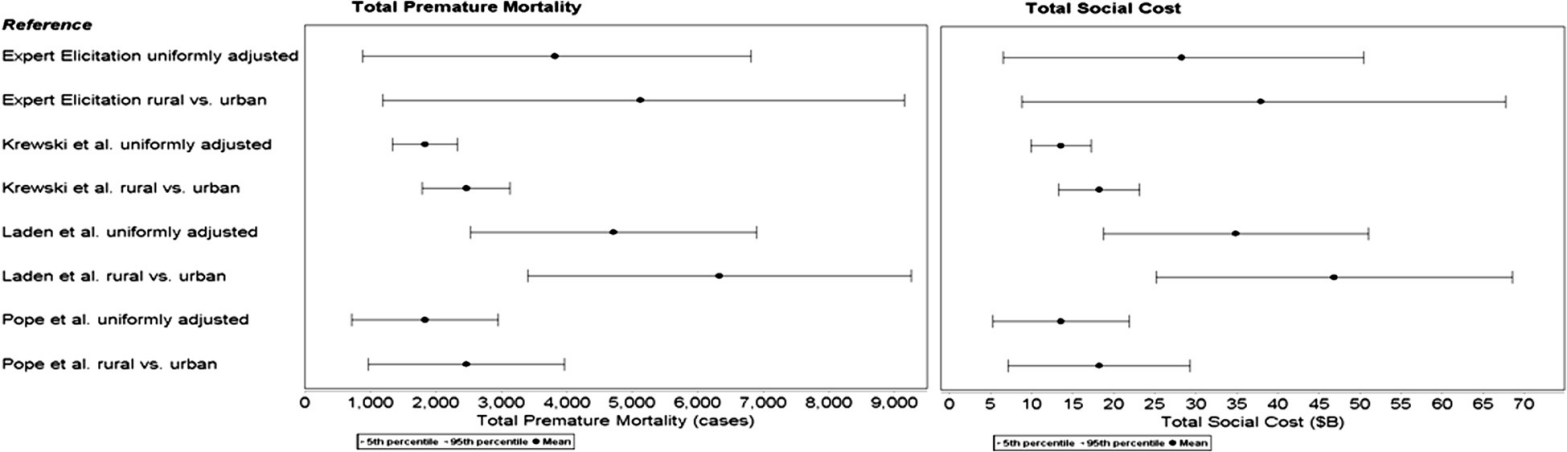
Incidence and total social cost associated with exposure to aromatic SOA from gasoline emissions.

Figure 2 presents a nationwide map of predicted premature mortalities attributable to aromatic hydrocarbons in gasoline associated with the expert elicitation concentration-response function. Table 5 and Figure 3 provide a summary of predicted premature mortality and monetized estimates of social cost based on all four different concentration-response functions. Predicted premature mortalities range from nearly 1,850 to more than 4,700 cases, depending on which concentration-response function is used, which correspond with approximately $13.6B to $34.9B in total social costs. The 5^th^ and 95^th^ percentiles from each study are included in the parentheses, and represent the effect of uncertainty in the concentration-response functions only (e.g., there are many potential sources of uncertainty, but only those associated with the concentration-response functions are captured in BenMap). Our recommended best estimate is approximately 3,800 premature mortalities based on the mean of the expert elicitation concentration-response function. Using the central estimates from the Krewski [41] and Laden [42] studies, respectively, results in a confidence interval of 1,800 to 4,700 for a central estimate.

**Table 5:** Premature mortality and total social cost for health impacts associated with exposure to SOA from aromatic hydrocarbons in gasoline in the lower 48 states

**Reference**	**Beta**	**Premature mortality (cases)**^**a**^	**Value of mortality reduction ($M)**^**a**^	**Premature mortality (cases)**^**b**^	**Value of mortality reduction ($M)**^**b**^
[43]	0.015	4714 (2533, 6897)	$34.9B ($18.7B, $51.0B)	6330 (3402, 9262)	$46.8 ($25.2, $68.5)
[12]	0.006	1833 (717, 2951)	$13.6B ($5.3B, $21.8B)	2462 (962, 3963)	$18.2 ($7.1, $29.3)
[42]	0.006	1833 (1335, 2332)	$13.6B ($9.9B, $17.2B)	2462 (1792, 3132)	$18.2 ($13.3, $23.2)
[44]	0.011	3816 (886, 6814)	$28.2B ($6.6B, $50.4B)	5125 (1189, 9151)	$37.9 ($8.8, $67.7)

Notes:

Value of mortality reduction = $7.4M per case in 2006$.

Beta = percentage change in mortality for a 1 μg/m^3^ change in PM_2.5 _concentration.

^(a)^ = uniform application of the 0.69 scaling factor to account for sources of aromatic emissions.

^(b)^ = rural areas adjusted by 0.69; 100% of aromatic emissions in urban areas assumed to originate from gasoline.

The results in columns 3–4 (a) in Table 5 have been adjusted by 0.69 to account for the fraction of aromatic emissions attributable to gasoline sources based on the 2005 National Emissions Inventory. However, it is possible that the fraction of aromatic emissions from gasoline could be higher in urban areas (although, as noted previously, Reff *et al.*[46] have shown that spatial patterns of emissions from other sources of aromatics such as solvent usage are similar to gasoline). To explore the potential impacts of this assumption, we adjust only those counties designated as rural counties [47] by 0.69 and assume that 100% of emissions in urban areas are derived from gasoline sources. The results are shown in the final two columns of Table 5 and in Figure 3. Predicted premature mortality increases to a little over 5,000, and based on the concentration-response function used, ranges from 2,400 to over 6,300.

**Figure 4:**
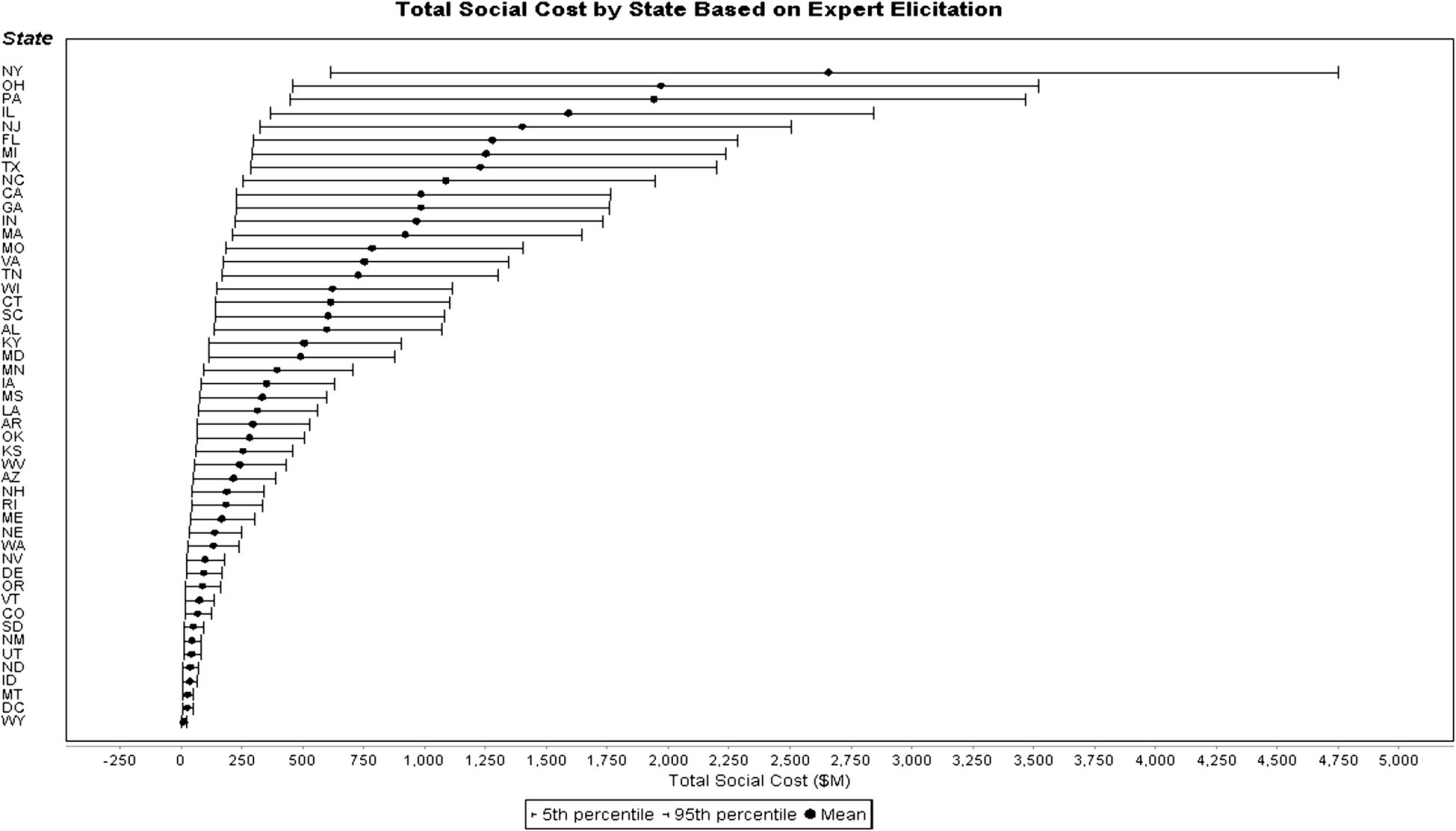
Total social costs by state based on expert elicitation concentration-response function.

Table 6 provides predicted premature mortalities and associated social costs for each of the four concentration-response functions. Figure 4 provides the results for each state, sorted from highest to lowest predicted impacts, using MetaDataViewer available from the National Toxicology Program [48] for the best estimate represented by the expert elicitation slope (the remaining results are proportional based on the results presented in Table 6; results not shown graphically). New York, with 0.21 μg/m^3^ of its PM2.5 attributable to aromatic SOA, shows the highest predicted impacts based on the number of exposed individuals. Ohio and Pennsylvania follow, with approximately 260 predicted premature mortalities each (based on the midpoint of the combined expert elicitation concentration-response function). The two states with the highest populations, Texas and California, are ranked eighth and tenth, respectively, for premature mortalities at approximately 170 and 130 expected cases, respectively.

**Table 6:** Predicted premature mortalities and associated social costs by state (Baseline Year = 2006)

**State**	**Population (2006)**	**Premature mortality based on expert elicitation **[43]** (cases)**	**Total social cost based on expert elicitation [**[43]**] ($M)**	**Premature mortality based on Krewski *et al. *2009 **[41]** (cases)**	**Total social cost based on Krewski *et al. *2009 ($M) **[41]	**Premature mortality based on Pope *et al. *2002 **[12]** (cases)**	**Total social cost based on Pope *et al. *2002 **[12]** ($M)**	**Premature mortality based on Laden *et al. *2006 **[42]** (cases)**	**Total social cost based on Laden *et al. *2006 **[42]** ($M)**
NY	11,721,250	359	$2,659	173	$1,277	173	$1,277	443	$3,278
OH	7,027,236	266	$1,972	128	$947	128	$947	329	$2,433
PA	7,856,478	263	$1,943	126	$933	126	$933	324	$2,395
IL	7,826,777	215	$1,592	103	$765	103	$765	266	$1,966
NJ	6,003,804	189	$1,402	91	$673	91	$673	234	$1,730
FL	12,353,717	173	$1,279	83	$615	83	$615	214	$1,581
MI	6,269,921	169	$1,254	81	$602	81	$602	209	$1,549
TX	13,969,855	166	$1,231	80	$592	80	$592	206	$1,526
NC	5,523,143	147	$1,090	71	$524	71	$524	182	$1,347
CA	22,483,409	133	$988	64	$475	64	$475	165	$1,221
GA	5,572,237	133	$986	64	$474	64	$474	165	$1,221
IN	3,915,380	131	$968	63	$465	63	$465	162	$1,196
MA	4,049,798	125	$922	60	$443	60	$443	153	$1,136
MO	3,608,441	106	$787	51	$378	51	$378	131	$972
VA	4,873,441	102	$754	49	$362	49	$362	126	$931
TN	3,822,406	99	$729	47	$350	47	$350	122	$902
WI	3,619,422	84	$624	40	$300	40	$300	104	$769
CT	2,253,322	83	$617	40	$296	40	$296	103	$761
SC	2,772,416	82	$605	39	$291	39	$291	101	$749
AL	2,927,474	81	$599	39	$288	39	$288	100	$742
KY	2,675,868	69	$507	33	$244	33	$244	85	$628
MD	3,715,953	67	$493	32	$237	32	$237	82	$610
MN	3,314,038	54	$397	26	$191	26	$191	66	$490
IA	1,906,272	48	$353	23	$170	23	$170	59	$435
MS	1,846,049	45	$335	22	$161	22	$161	56	$416
LA	2,630,768	43	$315	20	$151	20	$151	53	$391
AR	1,803,802	40	$296	19	$142	19	$142	49	$366
OK	2,233,442	38	$283	18	$136	18	$136	47	$350
KS	1,680,031	35	$257	17	$123	17	$123	43	$317
WV	1,187,545	33	$242	16	$116	16	$116	40	$298
AZ	3,911,781	29	$217	14	$104	14	$104	36	$269
NH	952,282	26	$190	12	$91	12	$91	32	$234
RI	653,356	25	$187	12	$90	12	$90	31	$230
ME	904,612	23	$169	11	$81	11	$81	28	$208
NE	1,058,917	19	$140	9	$67	9	$67	23	$172
WA	4,138,920	18	$135	9	$65	9	$65	22	$166
NV	1,603,777	14	$101	7	$49	7	$49	17	$125
DE	522,705	13	$95	6	$45	6	$45	16	$117
OR	2,383,414	12	$90	6	$43	6	$43	15	$111
VT	436,489	10	$77	5	$37	5	$37	13	$95
CO	2,974,597	9	$70	5	$34	5	$34	12	$87
SD	473,989	7	$53	3	$25	3	$25	9	$65
NM	1,300,700	6	$47	3	$22	3	$22	8	$58
UT	1,399,252	6	$45	3	$22	3	$22	8	$56
ND	413,558	5	$41	3	$19	3	$19	7	$50
ID	907,667	5	$38	2	$18	2	$18	6	$47
MT	639,955	4	$29	2	$14	2	$14	5	$35
DC	217,088	4	$28	2	$14	2	$14	5	$35
WY	347,896	2	$13	1	$6	1	$6	2	$16

## Discussion

Our best estimate of potential impacts is based on the expert elicitation concentration-response function recently endorsed by a US EPA Science Advisory Board Panel together with the regression-based adjustment factors to the CMAQv5.0 predictions, resulting in 3,800 predicted premature mortalities. This compares to a recent nationwide estimate of approximately 130,000 overall premature mortalities (for 2005) associated with all PM_2.5_ exposures recently discussed by Fann *et al.*[49] and based on the Krewski *et al.*[41] concentration-response function. The results presented in Fann *et al.*[49] were based on CMAQv4.7 together with additional monitoring data to estimate premature mortalities attributable to exposure to PM_2.5_ concentrations from all sources. The incremental contribution from exposure to aromatic SOA from gasoline using the adjusted results presented here and the Krewski concentration-response function represents approximately 1.4% of the total 130,000 estimated by Fann *et al.*[49]. While this may seem a small fraction of total PM-attributed mortality, these results are comparable to many public health measures, and with the Cross State Air Pollution Rule implementation in the next five years, are likely to constitute a higher portion of PM-related deaths in the future. Under this rule, if SO_2_ emissions decrease by an expected 50%, sulfate will become a smaller fraction of PM_2.5_; therefore, other sources will become more important, particularly since SOA from aromatic hydrocarbon precursors are not expected to decrease and could represent an increasingly larger fraction of exposures.

In addition to premature mortality, which dominates monetized estimates of total social cost, exposures to SOA from aromatics in gasoline are associated with other health outcomes, including exacerbation of asthma, upper respiratory symptoms, lost work days, and hospital emergency room visits.

To put our monetized social cost (central estimate of $28.2B) in context with other analyses, a recent study evaluated the public health impacts associated with exposure to direct emissions of PM_2.5_ attributable to congested traffic conditions [50] and estimated a total social cost of $31B (in 2007$). US EPA’s Heavy-Duty Highway Diesel Final Rule [51] estimates an 8,300 reduction in premature mortalities, a little more than twice the number of premature mortalities from this analysis.

While we have provided a best estimate of the public health impact of gasoline-driven aromatic SOA in PM2.5 based on current information, the exact sources of SOA remain a topic of ongoing debate. For example, a recent study in Los Angeles [52] found that gasoline emissions dominated SOA formation, accounting for nearly 90% of total aerosol formation, and the ratio of SOA to primary organic aerosol was approximately a factor of three. However, another recent study from California [53] found diesel exhaust to be responsible for 65% to 90% of vehicular-derived SOA and was 7 times more efficient at forming aerosol than gasoline exhaust. Overall, data suggest that across most areas in the U.S., SOA represents some 30%-40% of organic carbon concentrations [5,54-57].

Anthropogenic emissions have been shown to enhance biogenic SOA formation [58-60]. For example, SOA formation in the southeastern United States was investigated through a comparison with urban plumes in the northeast to identify biogenic versus anthropogenic precursors. The authors found that 70-80% of summertime carbon was of anthropogenic origin, and that anthropogenic precursors enhanced SOA formation from biogenic VOCs [60].

Photo-oxidation of aromatics has been shown to significantly contribute to anthropogenic SOA formation, but many factors contribute to variability in SOA formation that are not well understood, including spatial and chemical variability in emissions, the amount of time needed for PM formation, and varying ambient conditions at different scales [61,62]. CMAQ model performance of SOA formation has improved substantially with each version of the model, but likely doesn’t capture every process, given that SOA formation depends on varying atmospheric physical and chemical conditions which are simulated at coarser scales in the CMAQ model relative to the (unknown) scales at which they occur in the environment. For example, Snyder *et al.*[63] found markers for mobile and other sources differed by as much as 60% within the neighborhood scale and by greater than 200% within the urban scale.

The error in tracer-based estimates of aromatic SOA formation is approximately ±33% [64]; therefore, measurements are somewhat better understood than the specific processes and conditions leading to those observations. A strength of this analysis is the combination of modeling corroborated by empirical studies to provide a baseline estimate of predicted premature mortality associated with secondary organic particulate formation. Although aromatic SOA is a small component of PM2.5, our results show that exposure to secondary organic aerosol originating from aromatics in gasoline constitutes a non-trivial public health impact. Replacements for those aromatics are not risk-free themselves, and must be evaluated in a life-cycle context. As alternatives to aromatics in gasoline are contemplated, it will be important to consider the potential public health impacts associated with different transportation, fuel, and infrastructure design options (see, for example, Cook *et al.*[32], who developed a life-cycle assessment approach to evaluate the impacts of increased use of ethanol under several scenarios).

## Conclusions

Vehicle emissions of aromatic hydrocarbons originating in gasoline contribute to secondary formation of organic particulate matter. While many uncertainties exist in the exact mechanisms involved in secondary organic aerosol formation and the scales over which these mechanisms occur, our preliminary quantitative analysis provides a baseline estimate of premature mortality in the lower 48 states. Predicted premature mortalities range from 1800 to over 4700, demonstrating a non-trivial public health burden.

## Abbreviations

BenMAP: US EPA Benefits and Mapping Program v4.0; CB05: Carbon Bond 2005; CMAQ: Community Multiscale Air Quality modeling system; CMAQv5.0: Community Multiscale Air Quality model version 5.0; PM2.5: Fine particles less than 2.5 μm; NOx: Oxides of nitrogen; SOA: Secondary organic aerosol; VOC: Volatile organic compounds.

## Competing interests

The authors declare no competing financial interests. Funding for KvS, JB, and JS was provided by the restricted gift from Boyden Gray & Associates, PLLC to the Harvard Center for Risk Analysis. PVB participated as part of employment with the US EPA.

## Authors’ contributions

KvS wrote the manuscript with oversight from JS, JB conducted the BenMAP modeling using CMAQ results provided by PVB. All authors edited, read and approved the final manuscript.

## Supplementary Material

Additional file 1: Table S1 Comparison of Observed SOA Measurements and Unadjusted CMAQv5.0 Predictions. Click here for file

Additional file 2: Table S2 US EPA's SPECIATE Database Used to Determine the Fraction of Anthropogenic SOA from Aromatic Hydrocarbons in Gasoline. Click here for file
